# Chromatin Heterogeneity and Distribution of Regulatory Elements in the Late-Replicating Intercalary Heterochromatin Domains of *Drosophila melanogaster* Chromosomes

**DOI:** 10.1371/journal.pone.0157147

**Published:** 2016-06-14

**Authors:** Varvara A. Khoroshko, Viktor G. Levitsky, Tatyana Yu. Zykova, Oksana V. Antonenko, Elena S. Belyaeva, Igor F. Zhimulev

**Affiliations:** 1 Institute of Molecular and Cellular Biology, SB RAS, Novosibirsk, Russia; 2 Novosibirsk State University, Novosibirsk, Russia; 3 Institute of Cytology and Genetics SB RAS, Novosibirsk, Russia; Duke University, UNITED STATES

## Abstract

Late-replicating domains (intercalary heterochromatin) in the *Drosophila* genome display a number of features suggesting their organization is quite unique. Typically, they are quite large and encompass clusters of functionally unrelated tissue-specific genes. They correspond to the topologically associating domains and conserved microsynteny blocks. Our study aims at exploring further details of molecular organization of intercalary heterochromatin and has uncovered surprising heterogeneity of chromatin composition in these regions. Using the 4HMM model developed in our group earlier, intercalary heterochromatin regions were found to host chromatin fragments with a particular epigenetic profile. Aquamarine chromatin fragments (spanning 0.67% of late-replicating regions) are characterized as a class of sequences that appear heterogeneous in terms of their decompactization. These fragments are enriched with enhancer sequences and binding sites for insulator proteins. They likely mark the chromatin state that is related to the binding of cis-regulatory proteins. Malachite chromatin fragments (11% of late-replicating regions) appear to function as universal transitional regions between two contrasting chromatin states. Namely, they invariably delimit intercalary heterochromatin regions from the adjacent active chromatin of interbands. Malachite fragments also flank aquamarine fragments embedded in the repressed chromatin of late-replicating regions. Significant enrichment of insulator proteins CP190, SU(HW), and MOD2.2 was observed in malachite chromatin. Neither aquamarine nor malachite chromatin types appear to correlate with the positions of highly conserved non-coding elements (HCNE) that are typically replete in intercalary heterochromatin. Malachite chromatin found on the flanks of intercalary heterochromatin regions tends to replicate earlier than the malachite chromatin embedded in intercalary heterochromatin. In other words, there exists a gradient of replication progressing from the flanks of intercalary heterochromatin regions center-wise. The peculiar organization and features of replication in large late-replicating regions are discussed as possible factors shaping the evolutionary stability of intercalary heterochromatin.

## Introduction

Domain organization is essential for appropriate regulation of eukaryotic genomes. Many criteria, albeit not necessarily overlapping, have been used to identify *Drosophila* genomic domains: these include functional similarity, temporal and absolute levels of gene expression, replication timing, association with histone modifications and chromosomal proteins, chromatin accessibility, physical contacts, evolutionary conservation, topological associations, etc [1–14].

The very first domains in the chromosomes of diploid cells,—euchromatin and heterochromatin, were identified back in 1932 by Heitz based on their contrasting morphological features associated with distinct levels of chromatin packaging [15]. Next, large bands scattered over euchromatic arms of *Drosophila* polytene chromosomes and resembling pericentric heterochromatin in terms of chromatin packaging were described as intercalary heterochromatin (IH) [16] (see more in [17]).

Many remarkable features of IH regions suggestive of their unique organization were later discovered. For instance, IH regions were shown to complete replication very late and so they become underreplicated in endocycling polytene chromosomes [18–22]. Molecular borders of IH have been mapped and these were demonstrated to be highly stable across various types of somatic cells tested [23].

IH regions are typically quite large (100–600 kb), they display low gene density and long intergenic regions [24]. IH regions are composed of largely repressed chromatin [23,25], and encompass the genes with narrow tissue expression pattern (many of which are testis-specific) [4,21,24]. Recently, IH regions have been shown to correspond to topologically-associating domains [2,26].

One of the most intriguing properties of IH regions is their evolutionary conservation: they turned out to entirely or partially correspond to syntenic blocks where the number and the order of genes is maintained across distant *Drosophila* species [27]. Further, this paucity of chromosomal rearrangements within IH regions is accompanied with the higher degree of point mutations in IH-resident genes [28].

Clearly, many interesting facets of IH organization still await their discovery. The advent of "next-gen" technologies has provided us with molecular tools and approaches to analyze IH genome-wide in terms of chromatin protein composition [4,26], histone modifications [8], chromatin accessibility [10], and distribution of regulatory cis-elements [29,30].

In the present work, we performed further analysis of IH organization by combining the data obtained in the above-mentioned projects. This has allowed us to uncover somewhat unexpected heterogeneity of chromatin within IH. Namely, we observed significant enrichment of some types of IH fragments with enhancers and insulator-binding proteins; the borders of IH domains displayed features that were intermediate between the repressed chromatin of IH and the flanking actively transcribed regions encompassing house-keeping genes.

## Materials and Methods

### Map of chromatin types

Four basic chromatin types (aquamarine, lazurite, malachite, and ruby) used in the current study were defined earlier [26,31]. Each chromatin type was composed of 200 bp-long non-overlapping fragments belonging to the euchromatic portions of five large chromosome arms 2L, 2R, 3L, 3R, and X (total length 110715400 bp). Whole-genome map of four-state chromatin was processed as described in [31]. Chromatin types could not be assigned to a total of 5247600 bp. Whenever such sequence (gap) was at most 400 bp and was flanked by the same chromatin types on both flanks, the gap was annotated as having the same type as its flanks. Otherwise, the gap annotation was preserved. Series of consecutive fragments of the same chromatin type were merged together to define the positions of chromatin domains.

### RNA-seq data

RNA-seq transcriptome profiles were generated by the modENCODE project [32]. The profiles were downloaded from the FB2013_03 release of FlyBase, (http://flybase.org/static_pages/downloads/FB2013_03/genes/gene_rpkm_report_fb_2013_03.tsv.gz), and RPKM values were extracted for 29 tissues (FlyBase ID FBlc0000206, [33]) as well as S2 and Kc cell lines (FlyBase ID FBlc0000260, [34]). Each transcript was assigned with an RPKM value of the corresponding gene. The final table of RNA-seq data included RPKM values for 15294 genes. Of 27887 transcripts of protein-coding genes, we selected 18704 transcripts (13608 genes) for tissue RNA-seq expression data analysis.

For transcripts belonging to a certain chromatin type and for every tissue, a fixed threshold of RPKM values equal to three was applied. This threshold defined two classes of transcripts: one represented by silent genes (RPKM value ≤ 3) and the other one defined the group of expressed genes (RPKM value > 3). This threshold is consistent with the FlyBase database definition of genes having “Very low” (1 ≤ RPKM ≤ 3) or “No/extremely low” (RPKM = 0) expression (www.flybase.org).

### Mapping data for paused RNA polymerase II, insulators and enhancers

Stalled RNA polymerase II data (mapping of RNAs produced by promoter-proximal Pol II [35]) were downloaded from the GEO database, GSE18643 (samples GSM463298 and GSM463299).

Genome-wide data for insulator protein enrichment profiles were extracted from the modENCODE database (http://data.modencode.org/). In total, we used 16 tracks showing insulator protein distributions and 2 tracks showing enhancer localization [29]. The latter data were taken from S1 Table using Verification_status = “correct”, Positive = “1” tags. Data for the cis-regulatory modules (CRM) were downloaded from the RedFly resource (http://redfly.ccr.buffalo.edu/index.php) [36]. Description of the insulator protein and enhancer datasets is provided in S1 Table.

### Statistical tests

To estimate non-randomness of the observed distribution of chromatin types relatively to the localization of certain insulator or enhancer type, random permutation test was performed for each of the five chromosome arms, essentially as described earlier [31]. The only difference was that the number of overlapping chromatin fragments was inferred from the total length of overlapping chromatin fragments and insulators/enhancers. Length distributions of domains and inter-domain spacers were calculated for each chromatin type. Domains were named as An = {1, 2 …, N} and spacers were named as Sn = {1 (region from the chromosome start to the start of the first domain), 2, … N, N+1 (region from the end of the ultimate domain to the chromosome end)}. Index shuffling for An and Sn arrays was used to obtain random distribution of domains on the chromosome. Thus, the expected distribution of domains was generated, wherein the overlap with the insulators was totally random. In each iteration, the total length of domains belonging to the insulators was counted within the confines of each chromatin type. In total, we generated M ~ 10 [6] expected distributions and so we calculated the probability that the total length of the insulators in expected domain distribution {Randm} is above or below that of the observed one {Real}. These probabilities serve as estimates for the enrichment/depletion of the chromatin type within domains belonging to a chosen type of insulators. When the calculated P values were equal to zero or one (i.e. fewer/greater length observed for all expected distributions, respectively), the exact P value was calculated using normal distribution, as described in Boldyreva et al. [31]. Moving from the individual chromosomes to the whole genome, we ran permutation test simultaneously on all chromosomes as described in Boldyreva et al. [31] and computed overall estimates for the “whole genome” probabilities.

### Replication timing

Replication timing data for Kc cells [37] were downloaded from the ReplicationDomain site http://www.replicationdomain.com/. Positions of the probes and replication scores were selected by *awk* and recorded as a bed file. This file was then intersected with the domains by *intersectBed* from *bedtools* package [38] requiring at least 50% overlap. Replication timing scores of probes from each domain were imported into *R* package [39] and grouped in 0.5 steps by *hist* function. The densities of probes in each bin were plotted using *plot* and *points* functions.

The number of probes assigned to each chromatin type (based on at least 50% overlap) were 672971 (malachite), 510119 (lazurite), 381850 (aquamarine), and 1459467 (ruby). The probes belonging to each chromatin type were grouped together according to the replication timing score in 0.5 bins, and probe densities in each bin were plotted on the graph.

## Results

### Novel chromatin subtypes in IH regions

**Fig 1:**
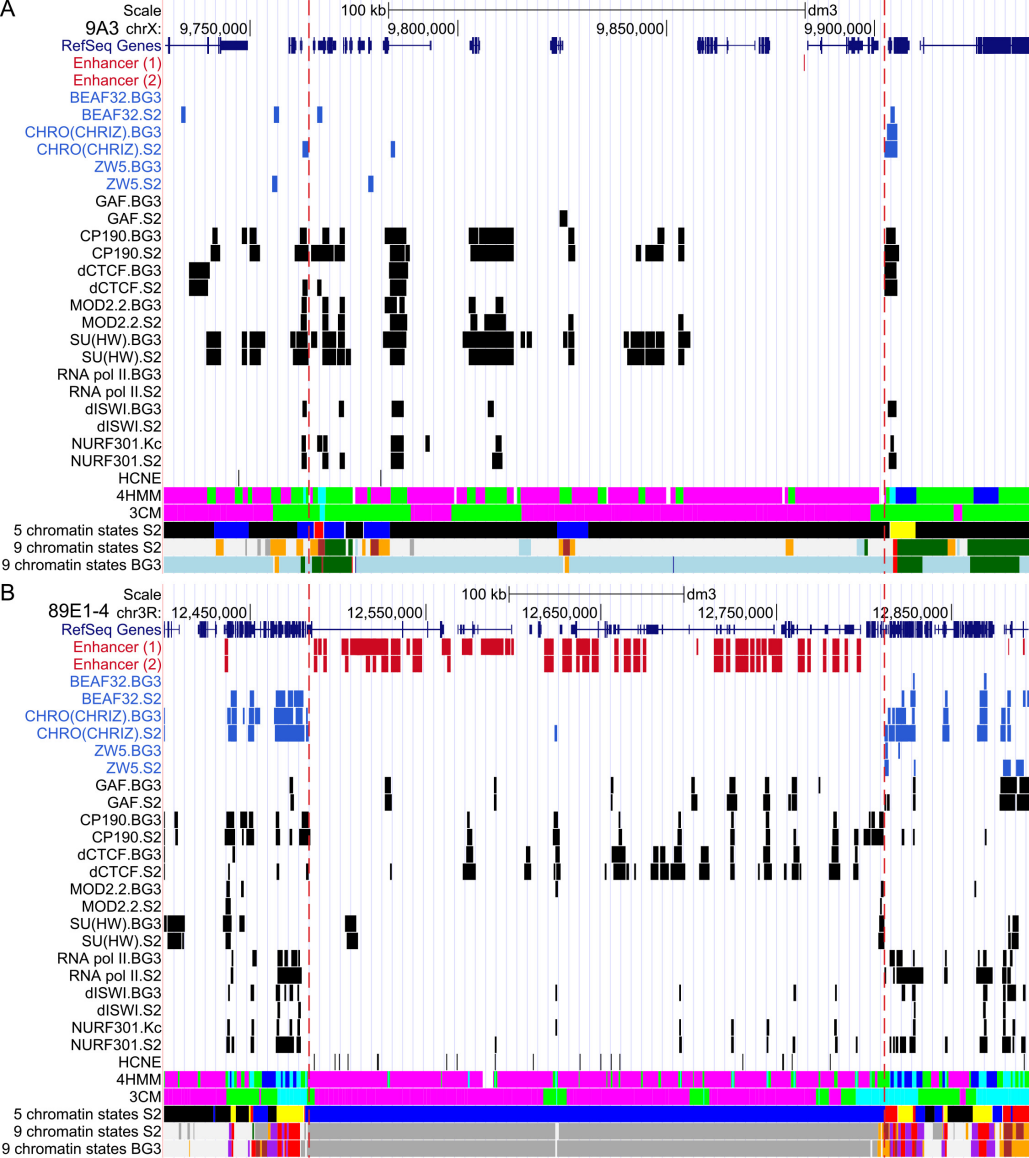
**Two representative chromatin passports for the IH regions 9A3 (A) and 89E1-4 (BXC) (B).** 4HMM map vs compactization map (3CM) by Milon et al., [10], 5 chromatin states by Filion et al., [4], 9 state map by Kharchenko et al., [8] and distribution of enhancer sites, sites of insulator protein binding, RNA pol II, remodeling factors dISWI and NURF301 (from modENCODE project, http://intermine.modencode.org/), positions of highly conserved noncoding elements (HCNEs) [3] are shown. Enhancers (1) and (2) are taken from RedFly [36] and Kvon et al. [29], respectively. Vertical red dashed lines delimit the edges of IH regions.

Top 62 largest IH regions previously reported as underreplicated in polytene chromosomes [18,20,21] were taken into analysis. Importantly, the size of underreplicated region is typically smaller than that of the matching IH band. Hence, we only used the underreplicated IH regions whose molecular borders were previously mapped: 60 regions described in Belyaeva et al. [23] with two more, 10А 1–2 and 10В 1–2, referenced in Zhimulev et al. [26]. The total span of these IH regions is about 12% of the genome total, and this value remains unchanged when other cell types are considered [23]. IH bands are invariably flanked with interbands, the regions that tend to harbor house-keeping genes [26]. Positions of genomic regions that this analysis focuses on are indicated on the molecular maps [23]; in the present work the borders of the regions used are shown as vertical red dashed lines (Fig 1 and Figs A-BH in S1 File). This allows matching the IH regions in polytene chromosomes and the physical map.

Heterogeneity of chromatin in IH regions was already apparent when looking carefully at the distributions of five basic chromatin types in the seminal work by Filion et al [4]: 97% of the combined length of IH regions were occupied by silent chromatin types BLACK and BLUE (84 and 13%, respectively); the remaining 3% corresponded to active chromatin types, RED and YELLOW. Somewhat higher value was observed, when IH regions were matched against active chromatin identified by Kharchenko et al. [8], in a study where finer partitioning of the genome into chromatin states was performed based on the combinations of histone modifications (more details are available in Belyaeva et al., [23]).

Here, we focused on the distribution and analysis of chromatin types that were identified using 4HMM by F. Goncharov in our group [26]. This model takes into account binding data for the "open chromatin" proteins and uses the data obtained for Kc, S2, BG3, and Clone8 cell lines as an input [30]. As a result, four basic chromatin types referred to as cyan, blue, magenta, and green have been identified [26]. In order to avoid possible confusion with color-coded chromatin types published by other groups, we had our chromatin types renamed as follows: aquamarine (formerly, cyan), lazurite (formerly, blue), malachite (formerly, green), and ruby (formerly, magenta). Preliminary inspection of these chromatin types clearly indicated that aquamarine was related to interbands, with notable enrichment for the interband-specific protein CHRO(CHRIZ) [40,41] and 5'-ends of house-keeping genes; lazurite chromatin matched gene bodies and morphologically corresponded to grey bands flanking the interbands; ruby chromatin corresponded to the repressed material of large dense bands; malachite chromatin showed little specificity in terms of the proteins enriched or morphological structures found in polytene chromosomes [26,31], so its role and organization await further research. Fragments of the four chromatin types described above are also present in IH regions. Our group has long been interested in IH, and we wanted to use these 4HMM data to gain further insight into how IH is organized.

**Fig 2:**
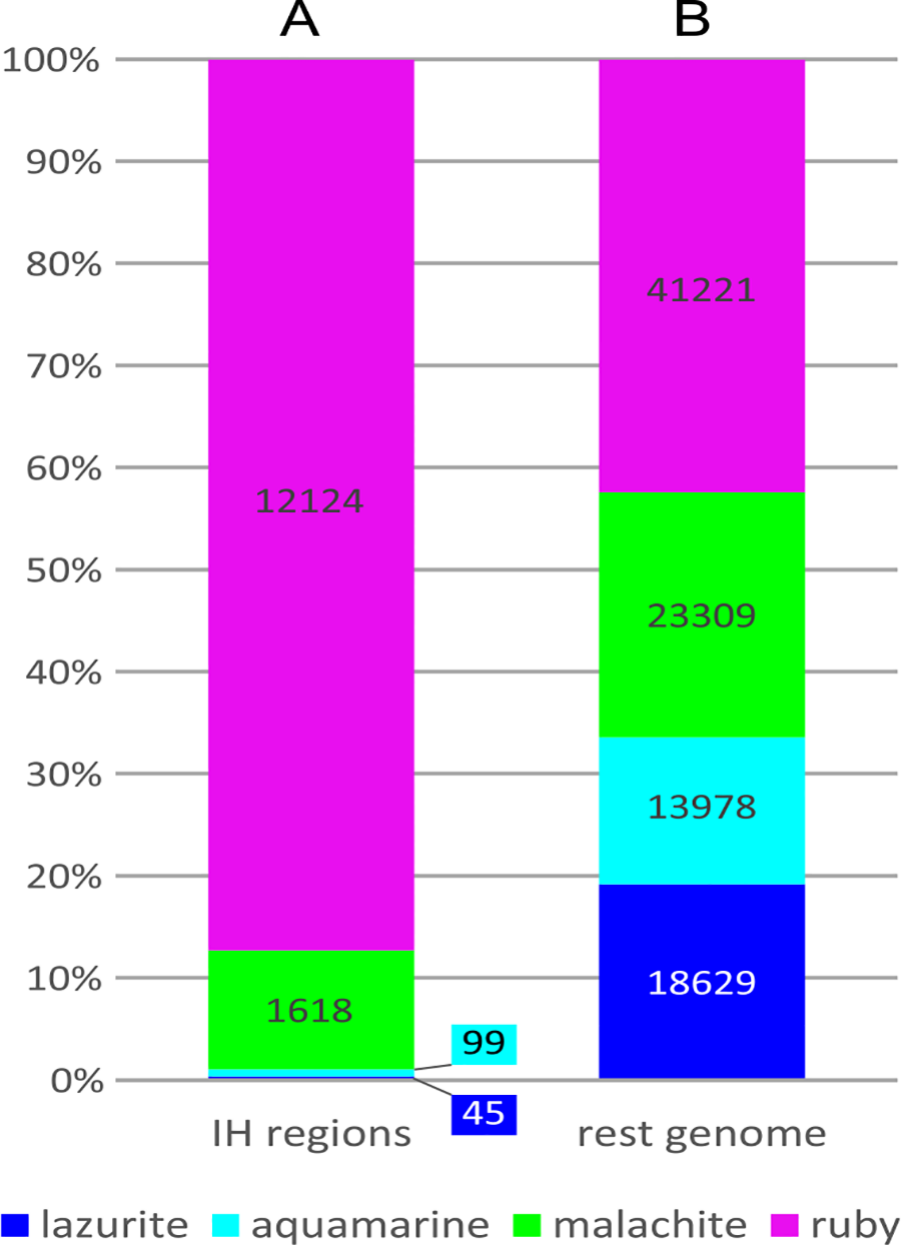
**Percentages of 4HMM-predicted chromatin types in IH regions (A) and genome-wide (B).** The length of each chromatin type (kb) is indicated inside the bars (the length of gaps was not taken into account).

The total length of the 62 IH regions selected is 14587 kb (i.e. 12.3% of the euchromatic part of the genome). The fractions of IH sequences occupied by the four chromatin types are 83.0% ruby, 11% malachite, 0.67% aquamarine, and 0.33% lazurite (5% are gaps) (Fig 2). Overall, ruby chromatin corresponds to the transcriptionally inert BLACK and BLUE chromatin types by Filion et al [4] (Fig 1).

Consequently, ruby chromatin shares all the characteristics of these chromatins types: low gene density, long intergenic intervals, late replication, underreplication in polytene chromosomes, presence of specific proteins (SUUR, D1, LAM). These domains contain functionally unrelated genes with narrow tissue and stage—specificity of expression. These characteristics are described in details earlier [4, 18, 20–25]. So, ruby chromatin largely defines the organization of IH regions. 4HMM model identifies ruby chromatin as repressed condensed blocks of chromosome material regardless of the very mechanism of repression.

The rest of the chromatin types,—malachite, lazurite and aquamarine,—do not show clear correspondence with the chromatin types and states proposed by Filion et al [4] or Kharchenko et al [8], so it was very interesting to study their organization in more detail. We created a "passport" for each of the 62 IH regions, which allowed us to compare the four chromatin colors with chromatin states and protein localization data available from the literature. Two representative "passports" are shown in Fig 1, with remaining 60 provided in the S1 File.

#### Malachite chromatin fragments in IH

We found 839 malachite fragments in the 62 IH regions (11% of their length). On average, one malachite fragment was 1618 bp. These fragments were further subdivided into internal malachite and border malachite subclasses. Border malachite regions are found in the transition zone between the ruby chromatin of IH bands and actively expressed regions of adjacent interbands (Fig 1).

**Fig 3:**
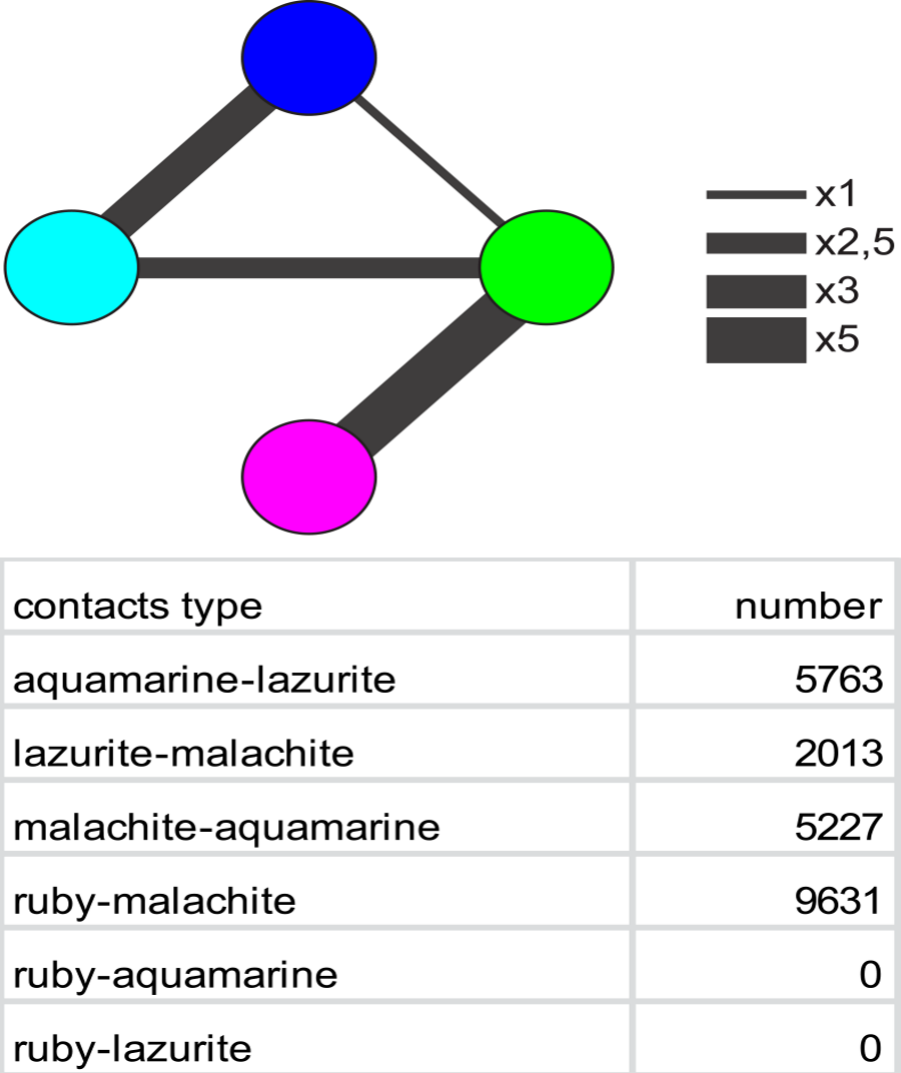
Contacts between the 4HMM states. The width of connecting lines is proportional to the frequency of contacts observed.

When calculating how often a chromatin type is bordered by other chromatin types, we observed that malachite chromatin is invariably found on the flanks of ruby domains regardless of whether the region analyzed belongs to IH or not. In fact, on the genome-wide scale, ruby chromatin was never directly bordered by either aquamarine or lazurite, with an intervening zone of malachite chromatin always found in between (Fig 3).

**Fig 4:**
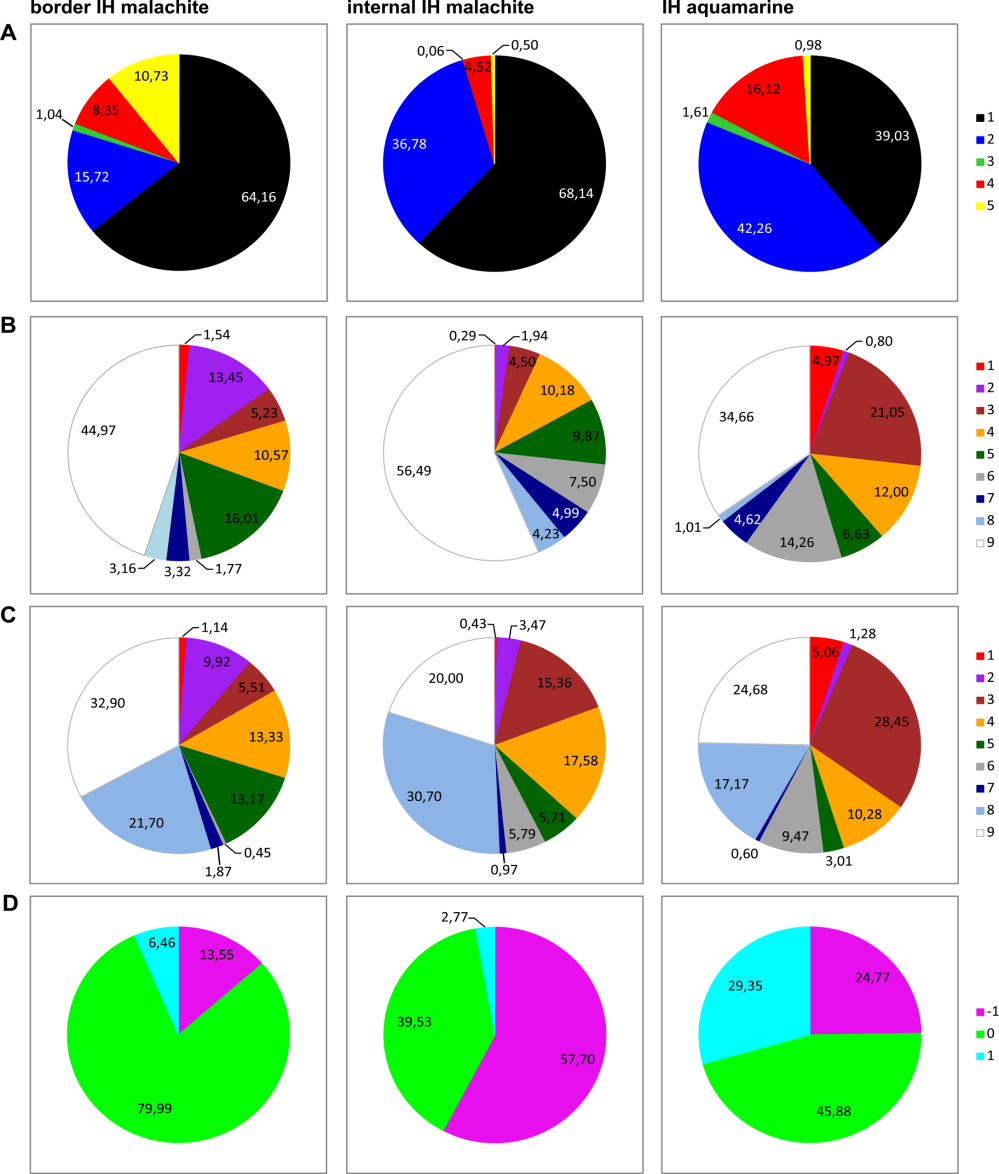
Overlap between 4HMM fragments and chromatin states and types. 5 principal chromatin colors reported in Filion et al., [4] (A); 9 chromatin states by Kharchenko et al., [8], S2 cells (B), BG3 cells (C); and 3 chromatin compactization classes [10] (D).

This puzzling observation prompted us to first analyze the correspondence between malachite chromatin and 9-state/5-type chromatin models proposed by Kharchenko et al. [8] and Filion et al. [4]. The results of this analysis are presented in Fig 4 as pie charts showing % overlap between border and internal malachite subclasses vs the above chromatin states and types. Most of the malachite chromatin fragments overlap with the repressed chromatin, as only 20% of border malachite and 5% of internal malachite sequences display overlap with the active RED and YELLOW chromatin types, with internal malachite and YELLOW chromatin types showing negligible overlap (Fig 4A).

The same trend is also seen when comparing our four chromatin types with the 9 chromatin states by Kharchenko et al. [8]. In S2 cells, about 30% of the border malachite fragments display overlap with the four active chromatin states, with notable enrichment of the state 2 (transcription elongation) (Fig 4B).

Internal malachite sequences and active chromatin states overlap by 16.5%, with notable lack of association with state 2, and the strongest association with state 4 instead. Both subclasses of malachite chromatin are virtually undetectable within active transcriptional start sites (TSS regions) (state 1). In BG3 cells, the numbers are slightly different but the trend is the same (Fig 4C). Thus, the malachite chromatin type is generally repressed, and the degree of this repression appears slightly lower in the border malachite than in the internal malachite regions.

Next, we proceeded to comparing the distribution of IH-embedded malachite regions against the “open” and “closed” regions, as defined by their resistance to DNaseI treatment, and which translates into the compactness of chromatin [10]. Three degrees of chromatin compactization (3CM model) have been described: 1 (“open”, active), –1 (“closed”, repressed), and 0 (“neutral”, intermediate). Internal malachite fragments show 58% overlap with closed chromatin, whereas border malachite displays 80% overlap with neutral chromatin (Fig 4D). The fraction of open chromatin was very low and totaled 3% for the internal malachite and ~6% for the border malachite. Thus, border and internal malachite regions are again distinct in terms of chromatin compactization, and the former chromatin subclass appears more open than the latter. Summarizing, the internal malachite appears more similar to the ruby environment it resides in, whereas border malachite is intermediate between ruby and adjacent transcriptionally active interbands.

**Fig 5:**
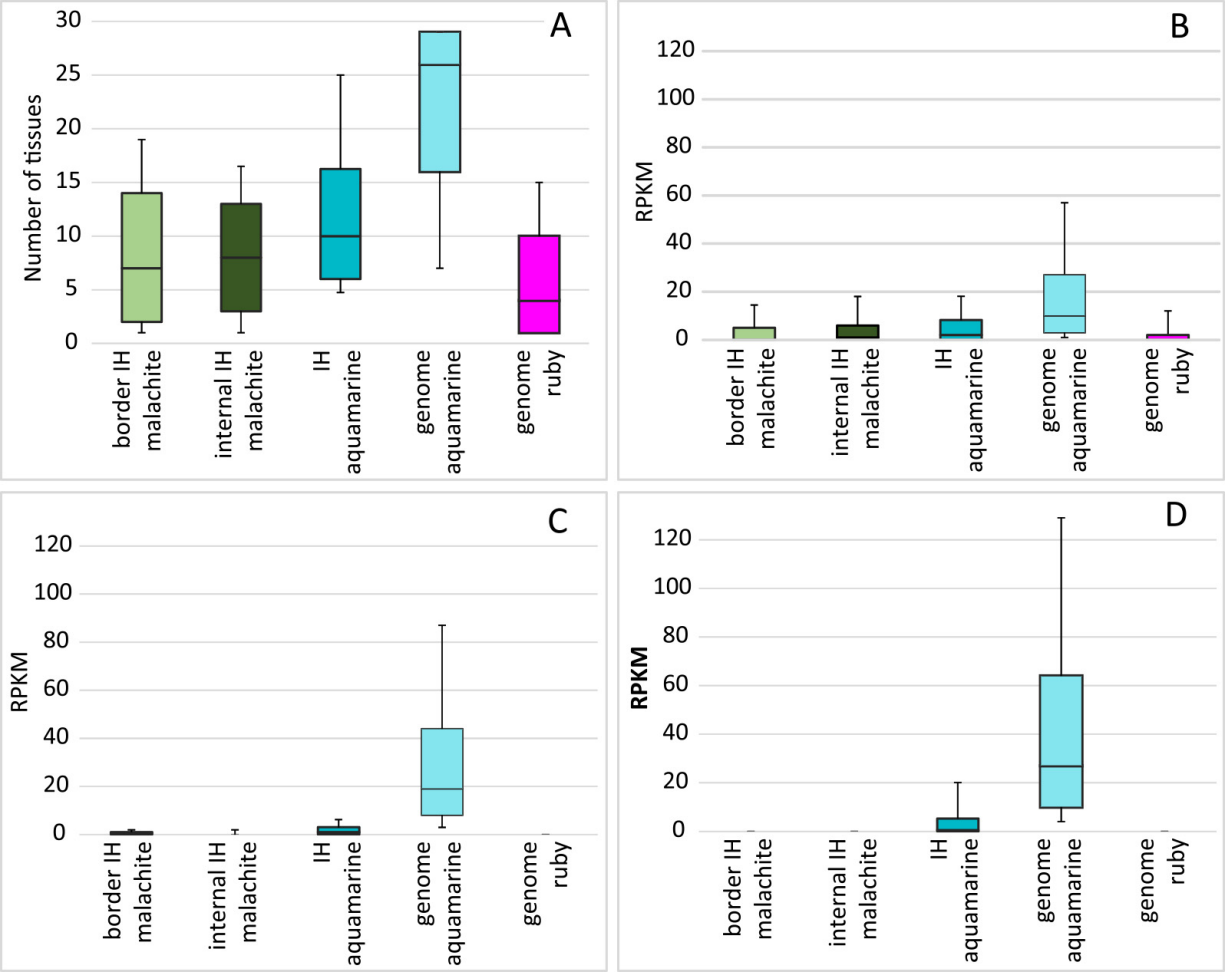
Gene expression in different 4HMM chromatin types. (А) The number of tissues where genes are active (RPKM>3). (B) Magnitude of gene expression summed for 29 tissues. Expression range in S2 (C) and Kc167 cells (D). Quartiles computed for the RPKM values are classified by the chromatin type. The distribution of the first, second and third quartiles of RPKM values for the datasets of transcripts are restricted by the chromatin types. For each chromatin color, the bottom part of the bar denotes the interval from the first to the second quartile; the top part denotes the interval from the second to the third quartile. In all panels, various chromatin types are shown on the X axis (from left to right: border malachite IH, internal malachite IH, internal aquamarine IH, aquamarine genome, ruby genome. Whiskers below/above the 1st/3rd quartile correspond to the 12.5th and 87.5th percentiles.

In order to understand whether the observed differences between the border and the internal malachite regions may correlate with the expression of genes they host, we estimated the breadth of gene expression, i.e. the number of tissues where these genes are active. To do so, we selected the genes whose TSS mapped within either of the chromatin subclasses were used. As it follows from the data presented in Fig 5A, both malachite subclasses are very similar in that they both comprise genes that are expressed in very few adult or larval tissues (median = 7–8 tissues). This is higher than what is observed for ruby genes genome-wide (median = 4), but is significantly lower than the typical value found for house-keeping genes that are active across the vast majority of *Drosophila* tissues (genome aquamarine). Thus, the genes from malachite chromatin are moderately expressed. We reached the same conclusions when using another metrics expressed as RPKM (expression profiling data summarized in 29 tissues by Graveley et al. [42]) (Fig 5B): both border and internal malachite genes display low expression levels intermediate between genome aquamarine and ruby. Hence, it is unlikely that the extent of chromatin decompaction is directly linked with transcription. This is particularly important when analyzing gene expression of malachite genes in S2 and Kc datasets (Fig 5C and 5D, respectively) as this analysis clearly shows that such genes are virtually silent in cell cultures.

#### Aquamarine chromatin fragments in IH

There are 104 aquamarine-type fragments totaling roughly 100 kb in 62 IH bands considered in our analysis (0.67% of the length of IH regions) with an average length of 1 kb (median = 800 bp). Stretches of aquamarine chromatin are present in 43 IH bands. Given that this chromatin type was originally discovered as characteristic of actively transcribed interband regions [26], we wondered what would be their possible function in the context of IH bands, where interbands are absent by definition. Visual inspection of these IH-embedded aquamarine sequences failed to reveal any correspondence with the published chromatin types. So, we looked into the overlap between aquamarine fragments and 9 chromatin states [8], 5 chromatin colors [4], chromatin accessibility [10], and binding sites for RNA pol II and remodeling factors dISWI and NURF301. As it turned out, 16% of aquamarine fragments overlapped with RED chromatin [4], the remaining 84% were found in BLACK and BLUE (Fig 4A). In S2 cells, 38% of aquamarine fragments overlap with states 1–4 (active chromatin states by Kharchenko et al., [8]), with notable enrichment for state 3 (implicated in gene regulation, «enhancer type») and state 4 related to state 3 (Fig 4B). The extent of overlap between aquamarine and state 3 is even higher when Bg3-derived datasets are considered (Fig 4C).

In terms of chromatin accessibility, the vast majority (70%) of IH-resident aquamarine fragments overlap with closed (–1) and neutral (0) chromatin [10], with only 30% matching the regions of open, nuclease-sensitive chromatin.

**Fig 6:**
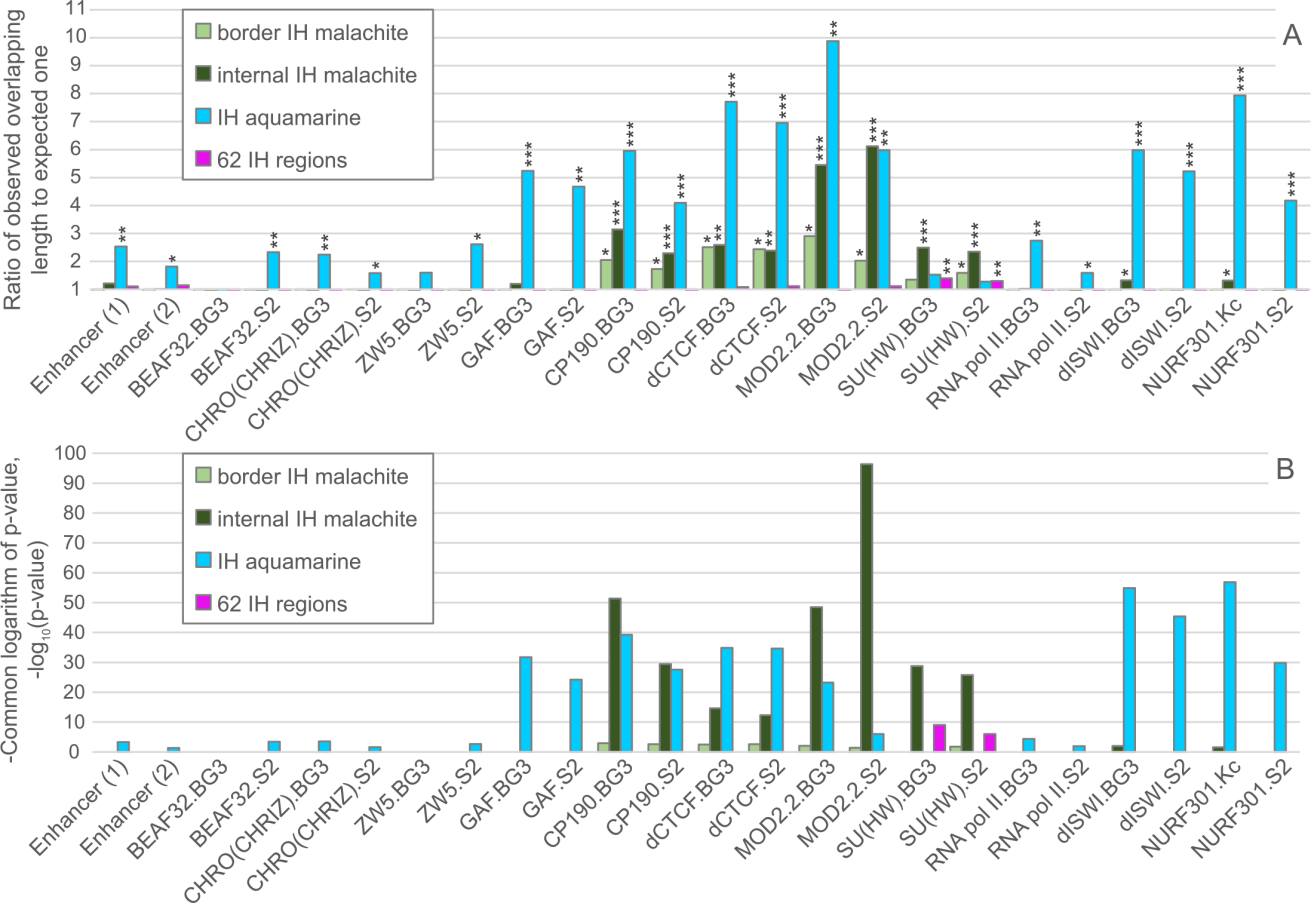
Enhancers and protein distributions across different 4HMM chromatin types. (A) Ratio of the observed fraction of overlapping fragments to the expected one. Observed fraction means the ratio of the total length of genomic regions associated with a protein of interest to the total length of the chromatin type in IH. Expected fraction is the fraction of overlap expected by chance (under random distribution model). Only the values above the “expected” threshold are shown. Asterisks denote probabilities of occurrence by chance *–p<0.05; **–p<1E^-3^; ***–p<1E^-25^. (B) Probability values that the observed overlap happened by chance. Bar height (-log_10_[P]) shows the significance levels for the enrichment of a chromatin type with regulatory elements or proteins indicated on the X axis. The probabilities were computed by the permutation Monte-Carlo test, as described in Materials and Methods section. Enhancers (1) and (2) are taken from RedFly [36] and Kvon et al. [29], respectively.

The genes whose TSS reside in IH-embedded aquamarine chromatin are expressed in more tissues (Fig 5A) and at higher levels (Fig 5B), than are ruby- and malachite-genes; yet, these values are significantly lower than expression levels observed for aquamarine-genes sampled genome-wide. Intriguingly, these genes are essentially silent in S2 and Kc 167 cells (Fig 5C and 5D) despite the enrichment for RNA pol II and particularly NURF301 and dISWI above the genome average (Fig 6).

**Fig 7:**
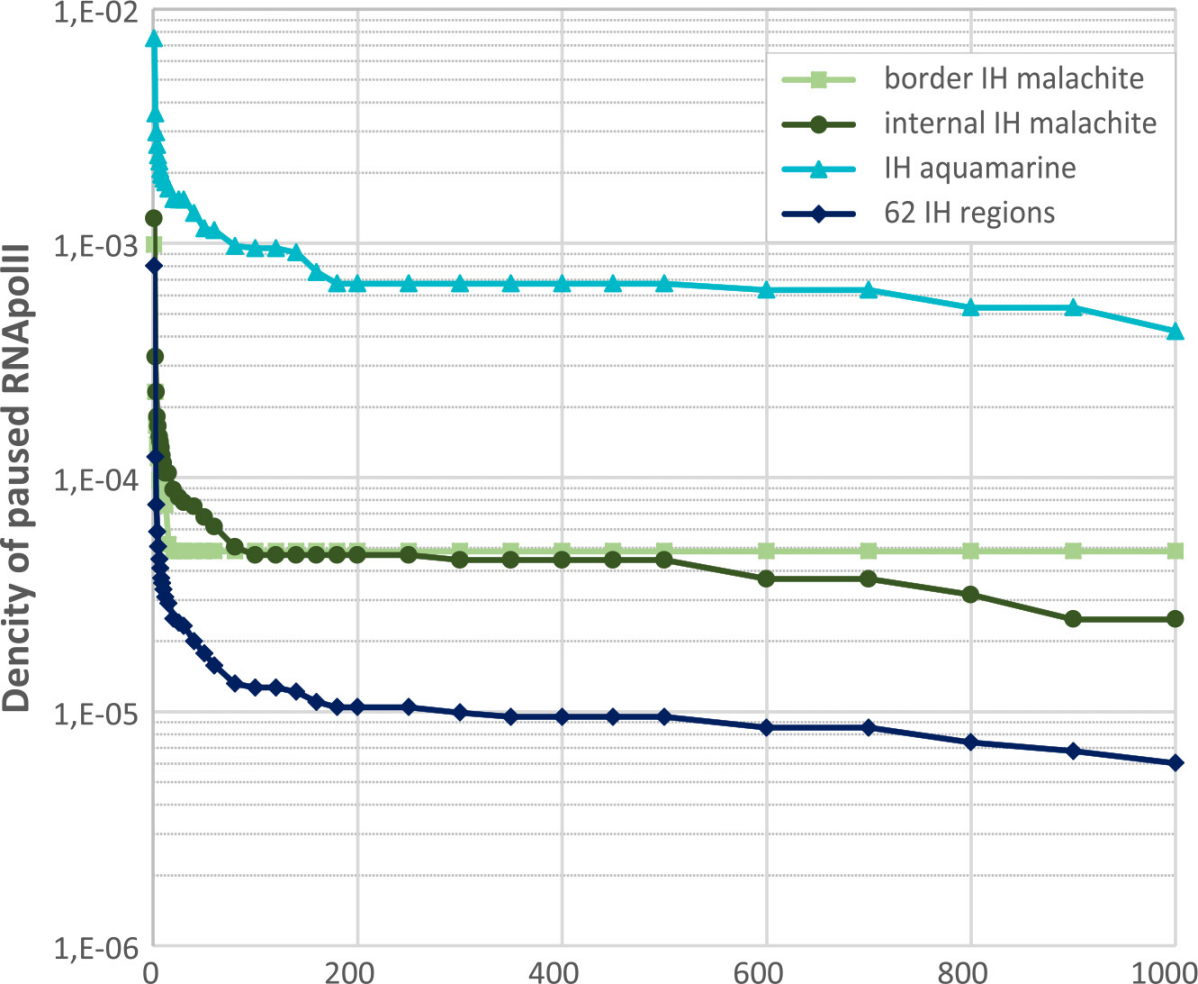
Enrichment levels of the paused RNA pol II in the four chromatin types. X axis shows the number of 5’-forward short non-polyadenylated transcripts (reads) produced by the paused RNA pol II (peaks). The density of peaks overlapping with a particular chromatin type is shown on the Y axis.

Low expression of genes residing in IH-embedded aquamarine in cell lines may be attributable to the fact that gene expression is tightly controlled at the early elongation step via RNA polymerase pausing mechanism. Indeed, the level of stalled RNA pol II is substantially increased in IH-aquamarine fragments. Fig 7 shows that the fraction of overlap between aquamarine fragments with stalled RNA pol II peaks is two orders of magnitude greater than the average value observed for IH regions in general or for the malachite chromatin.

These data make us conclude that IH-embedded aquamarine is a special chromatin type that shares many features of active chromatin except for the very transcription. Clearly, this fine chromatin subclass is detected by the 4HMM along with the interbands, despite the fact that interbands are transcriptionally active [26]. This suggests that aquamarine chromatin fragments in the genome are heterogeneous in terms of their genetic and epigenetic organization.

### Replication timing of various chromatin types found in IH

**Fig 8:**
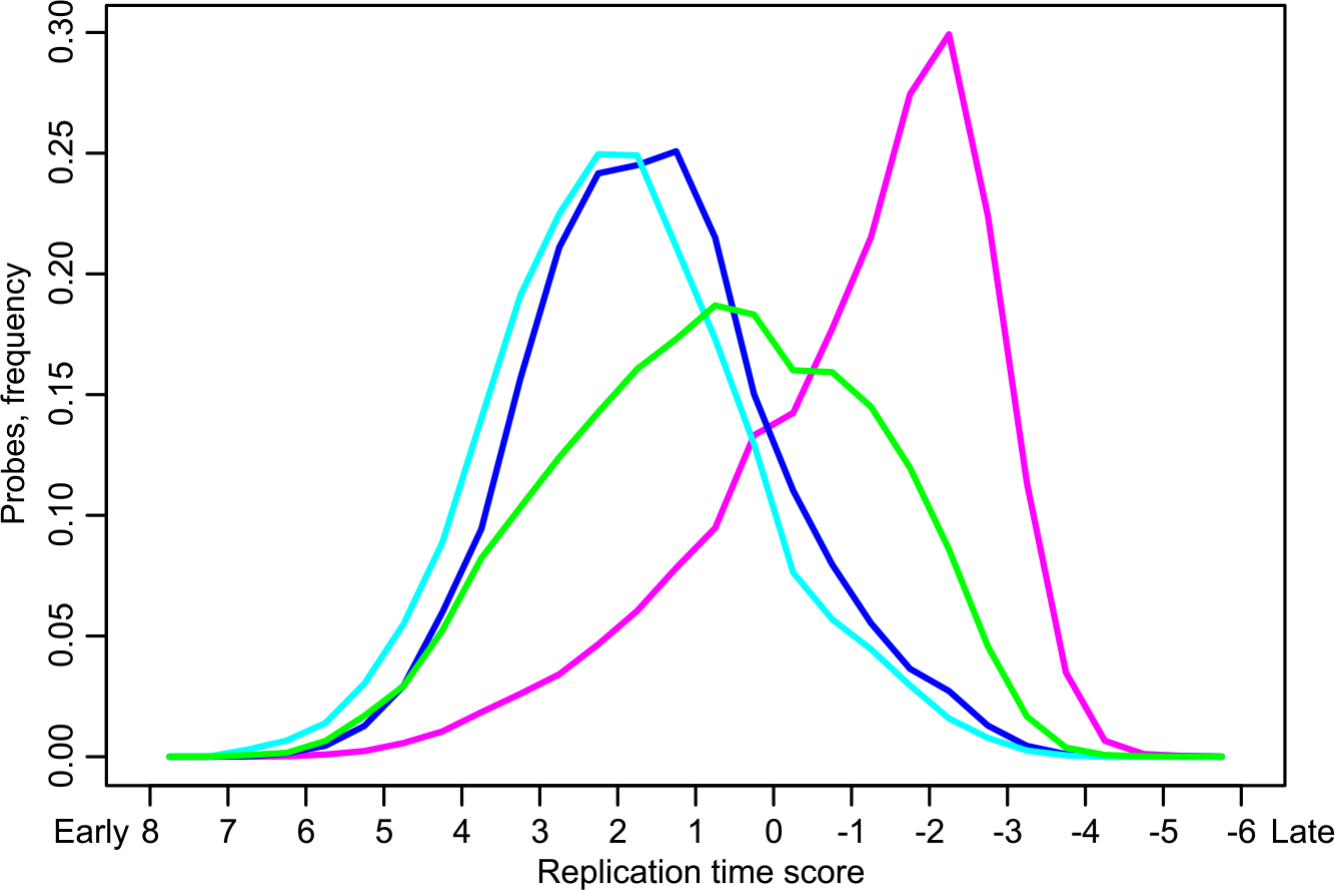
Replication timing of various 4HMM states measured genome-wide in Kc cells. Replication timing score is shown on the X axis with positive and negative values corresponding to early and late replication timing, respectively. The proportion of probes in each bin is shown on the Y axis.

First, we calculated replication timing of aquamarine, ruby, lazurite, and malachite chromatin types genome-wide. To do so, replication timing scores for Kc-167 cells were taken from Schwaiger et al. [37] and the number of probes reported as replicating early or late was calculated (see further details in Materials and Methods). As is shown in Fig 8 and much as expected, active chromatin represented by lazurite and aquamarine replicates early; repressed chromatin (ruby) replicates late. Malachite chromatin is again intermediate in terms of replication timing, as it replicates in the middle of S phase.

**Fig 9:**
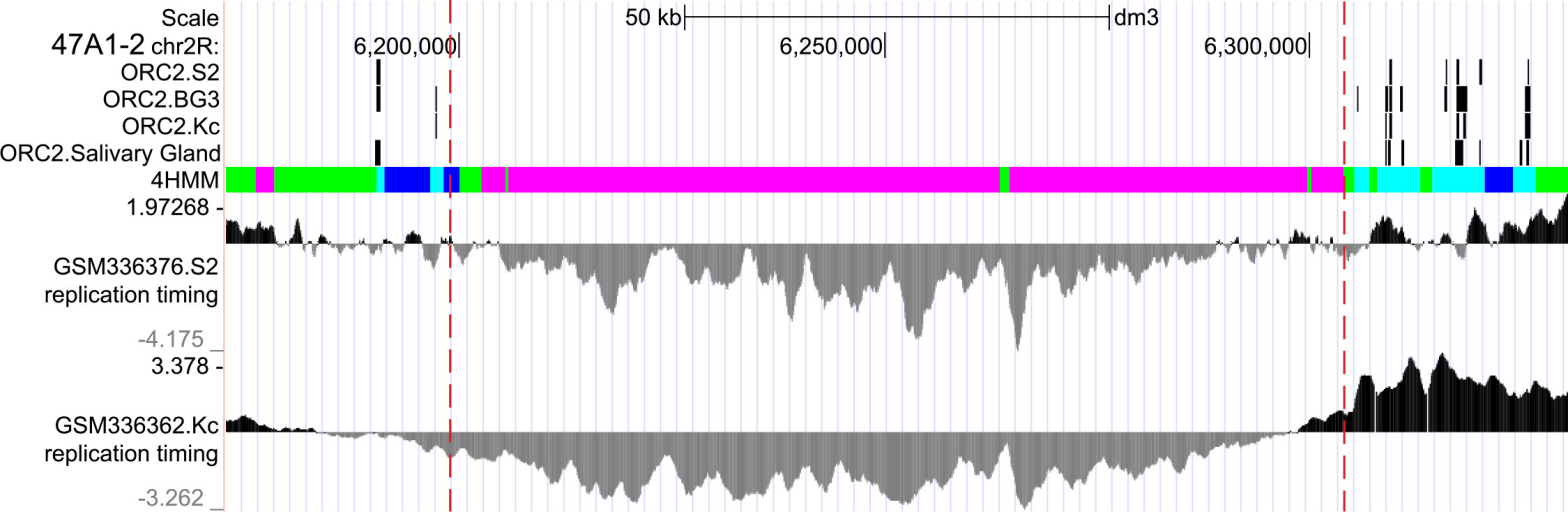
Replication timing gradient in the IH region 47A1-2. Comparison of positions of 4HMM-derived chromatin types with replication timing. Data on the distribution of ORC2 protein and replication timing were taken from the figures published in Belyaeva et al., [23].

Based on these data, replication profiles in IH regions were plotted. One example, (region 47А1–2), is shown in Fig 9 and illustrates a gradient of replication timing. Specifically, IH flanks replicate early, with replication becoming late towards the center of the IH band. Border malachite is consistently found within early-replicating flanks, and internal malachite replicates according to its position inside the band. The same holds true for the rest of the IH bands studied (appropriate replication profiles have been published elsewhere [23]). Thus, replication timing of a chromatin fragment is a function of its distance from the IH border.

### Distribution of regulatory elements in IH regions

IH regions were demonstrated to significantly overlap with conserved microsynteny blocks in drosophila [27]. Microsynteny blocks were proposed to function as regulatory domains, wherein expression of genes is under common control, which in turn precludes their separation by chromosomal rearrangements without loss of fitness [3,43]. In this regard, we were curious to see whether IH regions were peculiar in any way in terms of distribution of enhancer and insulator elements, both of which are known to mediate long-range effects.

#### Enhancers

Two sets of enhancer sequences were used in our analysis: embryonic enhancers [29] and enhancers annotated in the RedFly database [36]. The total length of these enhancer sequences across 62 IH bands, border malachite and IH-embedded aquamarine fragments was calculated. These values were appropriately normalized by the length of IH and corresponding chromatin subtypes to obtain enhancer density. In turn, the numbers obtained were compared to the enhancer density genome-wide, and the ratios above 1 were considered as evidence of enrichment (Fig 6A). IH regions are generally not enriched with enhancer sequences, and show density values comparable to the genomic average, border and internal malachite chromatin subtypes. In contrast, IH-embedded aquamarine shows moderate enrichment with enhancer sites. This conclusion is supported by the highly significant differences between the observed values and those obtained by random permutation (Fig 6B).

#### Insulators

We analyzed the distribution of seven well-annotated insulator proteins: BEAF32, CP190, dCTCF, GAF, MOD2.2, SU(HW), and ZW5. Additionally, CHRO(CHRIZ) protein was also incorporated into analysis, as it has recently been shown to be a subunit of BEAF32-containing protein complexes affecting the activity of BEAF32 and the establishment of long-range DNA contacts [44].

Visually, the distribution of enrichment regions for the above proteins is highly non-uniform across the IH regions. Peaks of BEAF32 and CHRO(CHRIZ) are largely restricted to the interbands that flank IH regions (an observation reported earlier in Zhimulev et al., [26]) with very few peaks observed inside IH. ZW5 appears depleted in IH, whereas the flanking interbands are depleted for MOD2.2 and SU(HW) (Fig 1 andFigs A-BH in S1 File).

Densities of insulator-bound sequences in IH, border and internal malachite, as well as in IH-embedded aquamarine have been quantitatively analyzed and are depicted in Fig 6. In general, IH bands show little if any enrichment for most of the insulator proteins analyzed. Statistically significant enrichment above the genomic baseline is only observed for SU(HW): (SU(HW)-VC.S2 p<10^−9^, SU(HW)-HB.BG3 p<10^−6^.

This is unlike the situation with IH-embedded malachite and aquamarine chromatin subtypes. CP190, dCTCF, MOD2.2, and SU(HW) are significantly enriched in internal malachite and border malachite, with particularly strong enrichment observed for MOD2.2 in internal malachite. Aquamarine fragments are generally rich in insulator protein binding sites: only 5 out of 104 IH-embedded aquamarine displayed no binding of insulator proteins.

These facts stimulated finer analysis of IH-embedded aquamarine fragments. We compiled the lists of insulator proteins found in each of the 104 aquamarine fragments (S1 Table), as this approach affords analysis of all protein combinations for each IH-embedded aquamarine fragment, individual IH regions and IH in general. The density of CP190, dCTCF, MOD2.2, GAF was 5–9 times above the genomic baseline, with a notable exception of SU(HW), which was not as overrepresented.

Several trends became immediately apparent when analyzing the combinations of insulator proteins. First, extensive co-binding of insulator proteins was observed. Only 20% of CP190 and 70% GAF sites are found located alone, the rest of the insulator proteins are present with at least one more partner. CP190/dCTCF is one of the most frequent combinations, whereas MOD2.2 is invariably associated with CP190 and SU(HW). Overall, these are the same combinations of insulator proteins as previously reported in the genome-wide analyses [45,46]. Extensive comparisons with these datasets are, however, of little power, because the sample size of insulators in aquamarine fragments is relatively small.

#### HCNE

IH regions are known to be home to many highly conserved non-coding elements (HCNE), most of which are regarded as regulatory [3,28,43,47]. Hence, we asked how they related to the 4MM chromatin types. Visually, no overlap between HCNEs and particular chromatin types or enhancers/insulators was evident (Fig 1 andFigs A-BH in S1 File).

## Discussion

IH regions are unique in many respects, including constitutive characteristics unrelated to epigenetic changes: they are long, display low gene density, large intergenic regions [18,24], and are enriched with HNCEs [3,28,43,47,48].

In the present work, using 4HMM we uncovered unexpected heterogeneity of chromatin composition in IH regions. Malachite and aquamarine chromatin regions were found scattered within IH. Malachite chromatin could be further subdivided into two subtypes, internal malachite (positioned centrally) and border malachite. Based on the data from other chromatin profiling studies [4,8,10], the latter subtype appears more open than the former. Notably, both subtypes of malachite chromatin harbor TSS of genes with generally very low expression in cell lines (Fig 5). This is important, as the 4HMM used in our study was based on the distribution of proteins in chromosomes of *Drosophila* cell lines, too. Hence, the apparent openness of border malachite is unlikely to be related to transcription. Malachite chromatin was also found enriched with insulator proteins CP190, SU(HW), dCTCF, and MOD2.2.

Of particular interest is the smallest group of IH-embedded chromatin regions, aquamarine (104 fragments spanning less than 1% of cumulative IH length). Despite its localization in repressive environment, this chromatin type appears fairly open. The genes whose TSS overlap with IH-aquamarine fragments are expressed at higher levels and in more tissues than other chromatin types present in IH. However, their expression is not as high as that of the genes whose TSS map to aquamarine fragments found elsewhere in the genome. IH-embedded aquamarine appears enriched with paused RNA pol II and nucleosome remodeling factors, as compared to the genomic baseline and other IH-resident chromatin types.

Even though the chromatin of genes whose transcription starts are found in IH-aquamarine appears relatively decompacted, these genes remain silent in S2 and Kc cell lines (in contrast to the aquamarine-genes from interbands that generally fall into the category of house-keeping genes). Possibly this is due to the absence of efficient elongation resulting from RNA pol II pausing [35,49].

These IH-embedded fragments of aquamarine chromatin stand out in that they are extremely rich in various enhancer and insulator elements and components (Fig 6). CP190, dCTCF, and GAF proteins are particularly abundant in IH-embedded aquamarine. 99 out of 104 IH-aquamarine fragments overlap with at least one region occupied by insulator proteins. Thus, aquamarine fragments may serve as bona fide markers of insulator protein binding sites visualized by the 4HMM. One can therefore speculate that the observed enrichment with insulator proteins is related to the open chromatin state characteristic of these regions. This position is consistent with a number of earlier experimental reports. For instance, targeting of CP190 was shown to result in the local decompaction of repressed chromatin, which was likely mediated by dCTCF. Intriguingly, this chromatin opening was not accompanied with activation of transcription [50]. Binding of CP190 was also shown to lead to nucleosome depletion ([51] for review), and Negre et al. [45] demonstrated pronounced nucleosome depletion in all insulator binding sites except for the SU(HW)-class.

Overall, IH regions are not particularly rich in insulators or enhancers. We did not observe pronounced association between HCNEs and the positions of enhancers/insulators. However, the enrichment itself may not be as important as once thought and the entire IH domain may rather be governed by the mutual arrangement and hierarchy of cis-regulatory elements and their long-range effects. We believe this idea may offer one of the explanations for the striking evolutionary conservation of IH regions.

Yet another important fact established in the present work deals with the mutual distribution of various chromatin types in IH regions. As it turned out, malachite chromatin invariably joins aquamarine and ruby chromatin types, and so it appears to function as a mandatory separating element. Border malachite subtype delimits ruby chromatin of bands and aquamarine chromatin of interbands; internal malachite in turn separates ruby chromatin from the IH-aquamarine subclass. It appears likely that malachite chromatin is a transitional type of chromatin between repressed and active chromatin types, and may function to counteract the spreading of inactive chromatin into neighboring regions (or the other way around).

Recently, it was shown that the borders of IH regions are the same in different chromosome types—diploid and polytene [23, 26], due to the permanent activity of neighboring interbands. Therefore, it is not surprising that flanks of IH regions adjacent to interbands always contain border malachite. However, inside the IH regions the distribution of subtypes of aquamarine and internal malachite evidently varies in different cells. At least two main reasons may account for this: tissue-specific activity of genes and tissue-specific distribution of binding sites of insulator proteins. This conclusion may explain the plasticity of chromatin organization in the chromosomes of different cell types.

One more aspect of domain organization of IH is related to its particular replication timing. IH regions have long been viewed as blocks of late-replicating genes that simultaneously enter replication. However, this position has been recently challenged. Replication origins are very scarce in IH bands and are predominantly found in the flanking interbands. Consequently, replication begins from the flanks and progresses inwards, so that two convergent replication forks form a single replication unit. The converging replication forks fail to meet before the end of the S period, and so a fraction of chromosomal fibers remain underreplicated. Stalling of replication forks is accompanied with formation of free DNA ends. This is interpreted as double-stranded DNA breaks by the cell machinery, and so these DNA ends are joined together [52] thereby forming deletions and other subchromosomal rearrangements. Originally, this scenario was proposed based on the cytological analysis of partial chromosomal rearrangements in polytene chromosomes upon blocked replication ([53] and references therein; [54,55] and has recently been confirmed directly at the molecular level [21]. The authors discussed the functional importance of underreplication as a factor of somatic instability that affects gene copy number in endocycling cells. So, it is clear that polytene chromosome cells "lose" much of the DNA during the very first cycles of replication, and this underreplication allows the cell to save time and energy in subsequent replication events. Also, replication of IH material progressing from the flanks center-wise results in a gradient of replication of IH-embedded genes, with earlier replication of sequences found on the flanks. This conclusion is supported by the replication profiles and replication timing studies (Figs 8 and 9). This gradient, in turn, may directly affect gene expression levels by changing the number of template DNA molecules.

It must be noted that underreplicated regions constitute a significant proportion of the euchromatic part of genome (19% according to Yarosh and Spradling, [21]) and so the gradient of replication (and underreplication) is an evolutionary advantageous trait that may contribute to the evolutionary stability of IH regions across *Drosophila* species. Clearly, the extent of underreplication and the time of replication completion correlate with the size of IH regions [21,23,56]. So, it is the large size of IH regions displaying late replication that likely defines their peculiar domain organization and underlies their evolutionary conservation.

## Supporting Information

S1 File **Figs A-BH in S1 File. Chromatin passports for IH regions.** 4HMM map vs compactization map (3CM) by Milon et al., [10], 5 chromatin states by Filion et al., [4], 9 state map by Kharchenko et al., [8] and distribution of enhancer sites, sites of insulator protein binding, RNA pol II, remodeling factors dISWI and NURF301 (from modENCODE project, http://intermine.modencode.org/), positions of highly conserved noncoding elements (HCNEs) [3] are shown. Enhancers (1) and (2) are taken from RedFly [36] and Kvon et al. [29], respectively. Vertical red dashed lines delimit the edges of IH regions.
(7Z) Click here for additional data file.

S1 Table Datasets of binding sites of insulator proteins and distant regulatory regions of genes (enhancers). Extracted from http://intermine.modencode.org/. Enhancers (1) and (2) are taken from RedFly [36] and Kvon et al. [29], respectively.
(DOCX)

S2 Table Overlapping of the aquamarine fragments with enhancers, binding sites of insulator proteins, RNA pol II, dISWI and NURF301. Enhancers (1) and (2) are taken from RedFly [36] and Kvon et al. [29], respectively.
(XLSX)
