# Supplementary material for: Chromatin Heterogeneity and Distribution of Regulatory Elements in the Late-Replicating Intercalary Heterochromatin Domains of Drosophila melanogaster Chromosomes
Source: PLoS One. 2016 Jun 14;11(6):e0157147. doi: 10.1371/journal.pone.0157147 (PMC4907538; doi:10.1371/journal.pone.0157147)
Supplement: S1 Table — Extracted from http://intermine.modencode.org/. Enhancers (1) and (2) are taken from RedFly [36] and Kvon et al. [29], respectively. (DOCX) [file pone.0157147.s002.docx]

| Track type  (insulator or enhancer) | Number of binding sites and regulatory regions | Total length (bp) |
| --- | --- | --- |
| Enhancer (1) | 5493 | 11019192 |
| Enhancer (2) | 3421 | 7250373 |
| BEAF32.BG3 | 528 | 678027 |
| BEAF32.S2 | 4616 | 10194137 |
| CHRO(CHRIZ).BG3 | 4783 | 12028867 |
| CHRO(CHRIZ).S2 | 4738 | 16373906 |
| ZW5.BG3 | 1203 | 2018856 |
| ZW5.S2 | 2705 | 4979794 |
| GAF.BG3 | 3928 | 9102732 |
| GAF.S2 | 3639 | 9338050 |
| CP190.BG3 | 3896 | 7189816 |
| CP190.S2 | 4319 | 7017246 |
| dCTCF.BG3 | 1644 | 3997464 |
| dCTCF.S2 | 1922 | 5073439 |
| MOD2.2.BG3 | 852 | 1274655 |
| MOD2.2.S2 | 1358 | 2763734 |
| SU(HW).BG3 | 4452 | 13916544 |
| SU(HW).S2 | 4166 | 12899185 |
| RNA pol II.BG3 | 4706 | 8992967 |
| RNA pol II.S2 | 6679 | 20983332 |
| dISWI.BG3 | 5595 | 10189919 |
| dISWI.S2 | 6060 | 11529619 |
| NURF301.Kc | 3354 | 5112775 |
| NURF301.S2 | 6136 | 13870096 |
